# Explicit Relationship Agreements and HIV Pre-exposure Prophylaxis Use by Gay and Bisexual Men in Relationships

**DOI:** 10.1007/s10508-022-02382-9

**Published:** 2022-08-08

**Authors:** James MacGibbon, Benjamin R. Bavinton, Kerryn Drysdale, Dean Murphy, Timothy R. Broady, Johann Kolstee, Angus Molyneux, Cherie Power, Heath Paynter, John de Wit, Martin Holt

**Affiliations:** 1 Centre for Social Research in Health, UNSW Sydney, Sydney, 2052 Australia; 2 The Kirby Institute, UNSW Sydney, Sydney, Australia; 3 New South Wales Ministry of Health, Sydney, Australia; 4 ACON, Sydney, Australia; 5 Australian Federation of AIDS Organisations, Sydney, Australia; 6 Department of Interdisciplinary Social Science, Utrecht University, Utrecht, The Netherlands

**Keywords:** Male couples, Men who have sex with men, HIV PrEP, HIV prevention, Australia

## Abstract

Relationship agreements are important for HIV prevention among gay and bisexual men (GBM) in relationships, with research earlier in the HIV epidemic often finding that agreements specified monogamy or condom use with casual partners. There is evidence that HIV pre-exposure prophylaxis (PrEP) has shifted sexual practices among some men in relationships, such as allowing condomless sex with casual partners, but there has been little attention paid to relationship agreements among GBM who use PrEP. In this paper, we analyzed national, Australian, cross-sectional data from an online survey completed by non-HIV-positive GBM in 2021 (*N* = 1,185). Using logistic regression, we identified demographic characteristics, sexual practices and the types of relationship agreement that were associated with PrEP use among GBM in relationships. Using Pearson’s chi-squared tests, we explored whether PrEP users in relationships reported similar sexual practices to PrEP users not in relationships. PrEP use among GBM in relationships was independently associated with older age, identifying as gay, being in a non-monogamous relationship, having a spoken (explicit) relationship agreement, having a primary HIV-negative partner taking PrEP or a primary partner living with HIV, reporting recent condomless casual sex, reporting an STI diagnosis in the past year, and knowing at least one other PrEP user. We found that PrEP users in relationships had similar sexual practices to PrEP users not in relationships. GBM in relationships who have casual sex and who meet PrEP suitability criteria may be good candidates for PrEP. Our findings suggest that explicit relationship agreements remain important for HIV prevention, and they support PrEP use among GBM in relationships.

## Introduction

HIV pre-exposure prophylaxis (PrEP)–the regular use of antiretroviral medications–is highly efficacious at preventing HIV (Fonner et al., 2016; Molina et al., 2017). Since its approval by the US FDA in 2012, PrEP has been rapidly rolled out in some high-income countries, primarily targeted at gay and bisexual men (GBM) and other men who have sex with men (Bernays et al., 2021). In Australia’s most populous states (Victoria and New South Wales), 33% of non-HIV-positive GBM used PrEP in 2021 (Broady et al., 2021; Chan et al., 2021). Overall, the proportion of GBM in Australia protected by one or more combination prevention methods increased to a record high of 75% in 2019, with PrEP use overtaking condoms as the most common prevention method used by GBM with casual partners (Holt, Broady, et al., 2021).

As PrEP uptake has increased, researchers have turned their attention to its potential benefits for GBM in relationships, particularly those in non-monogamous relationships (Mitchell & Stephenson, 2015), and HIV-negative men in serodiscordant relationships who are concerned about HIV transmission (Starks et al., 2019). The existing literature on PrEP and GBM in relationships has used qualitative interviews and survey data to explore potential barriers and enablers of PrEP use, particularly in the USA (e.g., Gamarel & Golub, 2015; Grov et al., 2021; Hoff et al., 2015; Saberi et al., 2012; Starks et al., 2019). Some research has reported changes to sexual practices among men in relationships after commencing PrEP. For example, an Australian cohort study of GBM found that men in relationships who initiated PrEP were much more likely to have a partner who also used PrEP, to have casual sex, and to report receptive condomless sex compared with non-PrEP-users (Bavinton et al., 2021). Other research has found that PrEP users tend to report more sexual partners and more condomless sex than GBM who do not use PrEP (e.g., MacGibbon et al., 2021; Traeger et al., 2018). However, it has not been a focus of previous research to ascertain whether GBM in relationships who use PrEP have similar sexual practices to PrEP users who are not in relationships; that is, it is possible that PrEP users in relationships may prioritise sex with their primary partner and have fewer casual partners than single PrEP users, or conversely PrEP use may facilitate a broader range of sexual practices for GBM in relationships. Lastly, there has been little published research on the potential impacts PrEP may have on relationship agreements. Prior to PrEP, research has generally found that most Australian GBM in relationships either had agreements that prohibited casual sex or specified that condoms should be used with casual partners (Crawford et al., 2001; Kippax et al., 1997). After PrEP became more commonly used, national, Australian survey data showed that GBM in relationships who used PrEP were much more likely than non-PrEP-users to have condomless sex both within and outside the relationship, and to have agreements that permitted condomless sex with casual partners (MacGibbon et al., 2020). However, there was considerable variation in the types of agreement and sexual practices used by GBM after PrEP roll-out in Australia, with a notable minority either having no agreement with their relationship partner or appearing to have casual sex that was not permitted by their agreement (MacGibbon et al., 2020). Such agreements, or lack thereof, suggested that some gay men may experience difficulties in renegotiating old agreements, or initiating new ones that incorporate PrEP.

There is a well-established literature on GBM’s relationship agreements and HIV prevention (e.g., Hoff & Beougher, 2010; Hoff et al., 2010; Hosking, 2013; Parsons et al., 2013). Some studies have suggested that communicating about PrEP with a partner may be difficult (e.g., Bosco et al., 2021; Stephenson et al., 2021, 2022), yet no studies have addressed the practical details involved with negotiating PrEP use within a relationship. For example, how to broach the topic of PrEP with a partner and reach an agreement that one or both partners will take PrEP, and the type of sex that is allowed in and outside the relationship if a partner is using PrEP. For men who were already taking PrEP when they met their partners, it is also unclear from the existing literature how they raise ongoing PrEP use or discuss stopping PrEP; such decisions could form part of spoken relationship agreements to be monogamous, rules to protect couples against other STIs, or acceptable forms of casual sex and how to manage sexual health. Existing research does however suggest that the decision to use PrEP (or keep using it) is often an extension of an agreement about non-monogamy (Malone et al., 2018), and consequently, discussions between primary partners may encounter the same difficulties that many GBM experience discussing sex and non-monogamy (see Bonello, 2009). For monogamous couples, suggesting or raising the issue of PrEP may be particularly challenging, and signal mistrust or potential infidelity (Bosco et al., 2021; Malone et al., 2018; Quinn et al., 2020). There is some evidence that introducing PrEP to a relationship may have positive effects, but this may require partners to be confident discussing PrEP and related matters with their primary partners (Stephenson et al., 2021, 2022). One recent US-based study found that GBM in relationships who used PrEP were more likely to have a negotiated sexual agreement (open or closed) rather than no agreement, compared to men who did not use PrEP (Stephenson et al., 2022). This research found that GBM who were more comfortable talking to their partner about PrEP were also more likely to be using it (Stephenson et al., 2022). The ability to raise and negotiate an agreement is therefore likely to affect PrEP use among men in relationships, and this may need to be addressed by researchers, clinicians and educators who work with GBM.

In summary, the existing literature suggests that in addition to perceived risk of HIV, PrEP adoption within relationships is likely to be impacted by GBM’s capacity to form relationship agreements; and may be more likely for partners who are non-monogamous and can negotiate a spoken (explicit) agreement, rather than having no agreement or an unspoken/assumed agreement. Using national Australian, cross-sectional survey data, we therefore sought to explore the type of relationship agreements associated with PrEP use among GBM in relationships. Because of the lack of empirical data on this topic, we also sought to establish whether PrEP users in relationships reported similar sexual practices with casual partners to PrEP users not in relationships, and GBM not using PrEP. In doing so, the current study sought to understand how relationship agreements and the type of sex that GBM have were associated with PrEP use, to identify the specific education and health promotion needs of GBM in relationships who are considering or already using PrEP.

## Method

### Study Design and Participants

Data were collected as part of the PrEPARE Project, a repeated, cross-sectional study of Australian GBM’s attitudes to HIV prevention (Holt et al., 2022, 2021a, 2021b; MacGibbon et al., 2021). This analysis focuses on the 2021 round: a national, online, cross-sectional survey of GBM conducted in April–June 2021 using Qualtrics software (Provo, UT). The survey was promoted through community organisations, Facebook groups about HIV prevention, and paid advertisements on Facebook and the dating/hook up app Grindr. Potential participants were directed to the project website where study information and a link to the survey was provided. Participants were asked to indicate consent at the start of the survey. In 2021, eligible participants were aged 16 years or older, did not identify as female, did not identify as heterosexual, and lived in Australia (i.e., male or non-binary and gay, bisexual or queer-identified people could participate). The study was approved by the ethics committee of UNSW Sydney (HC16954) and endorsed by the community organisation ACON (2017/04).

### Measures

The questionnaire measures have been previously described (Holt et al., 2022; MacGibbon et al., 2019). We collected data on demographics, health and well-being, relationships with male and female partners, recent sexual practices, HIV status, and attitudes to and use of HIV prevention, including HIV pre-exposure prophylaxis (PrEP). The variables included in the analyses and their categories are shown in Table 1 and Table 2.

**Table 1:** Participant characteristics, sexual behaviour and risk reduction practices of non-HIV-positive men by PrEP use and relationship status, 2021

	Total	Non-PrEP-users		PrEP users		Test statistic	*p* value
		No relationship	Relationship	Relationship	No relationship		
	*N* = 1,185	*n* = 436	*n* = 277	*n* = 221	*n* = 251		
Median age (IQR)	37.0 (29.0–50.0)	35.0 (26.0–50.5)	36.0 (28.0–51.0)	40.0 (33.0–52.0)	36.0 (30.0–48.0)	19.05	< .001
Sexuality						110.3	< .001
Gay	951 (80.3)	283 (64.9)	245 (88.4)	210 (95.0)	213 (84.9)		
Bi/queer/other	234 (19.7)	153 (35.1)	32 (11.6)	11 (5.0)	38 (15.1)		
No. of male partners in last 6 months						264.86	< .001
None	144 (12.2)	96 (22.0)	34 (12.3)	4 (1.8)	10 (4.0)		
1–5	587 (49.5)	238 (54.6)	193 (69.7)	78 (35.3)	78 (31.1)		
6–10	198 (16.7)	50 (11.5)	30 (10.8)	50 (22.6)	68 (27.1)		
> 10	256 (21.6)	52 (11.9)	20 (7.2)	89 (40.3)	95 (37.8)		
CAIC						309.72	< .001
No casual male partner(s) or no anal sex	411 (34.7)	176 (40.4)	179 (64.6)	29 (13.1)	27 (10.8)		
Condoms only	146 (12.3)	85 (19.5)	31 (11.2)	13 (5.9)	17 (6.8)		
Any condomless sex	628 (53.0)	175 (40.1)	67 (24.2)	179 (81.0)	207 (82.5)		
Time since last test for STIs						272.24	< .001
≤ 12 months	777 (65.6)	214 (49.1)	126 (45.5)	201 (91.0)	236 (94.0)		
> 12 months	246 (20.8)	121 (27.8)	107 (38.6)	14 (6.3)	4 (1.6)		
Never tested for STIs	162 (13.7)	101 (23.2)	44 (15.9)	6 (2.7)	11 (4.4)		
STI diagnosis in last 12 months	259 (21.9)	57 (13.1)	19 (6.9)	74 (33.5)	109 (43.4)	142.04	< .001
Often/always serosorting	382 (32.2)	104 (23.9)	41 (14.8)	101 (45.7)	136 (54.2)	126.26	< .001
Often/always PrEP sorting	366 (30.9)	75 (17.2)	30 (10.8)	122 (55.2)	139 (55.4)	222.20	< .001
Often/always UVL sorting	104 (8.8)	28 (6.4)	13 (4.7)	28 (12.7)	35 (13.9)	21.34	< .001
Drug-enhanced sex in last 6 months	167 (14.1)	49 (11.2)	18 (6.5)	47 (21.3)	53 (21.1)	35.75	< .001

Data are median (IQR) or n (%). Tests of statistical significance are Kruskal–Wallis (for age) and Pearson’s chi-squared. CAIC = condomless anal intercourse with casual partners

**Table 2:** Factors associated with PrEP use among GBM in relationships in Australia, 2021

	All	Non-user	PrEP user	OR (95% CI)	*p* value	aOR (95% CI)	*p* value
	*N* = 498 (%)	*n* = 277 (%)	*n* = 221 (%)				
*Age*							
< 30	104 (20.9)	78 (28.2)	26 (11.8)	Ref		Ref	
30–39	154 (30.9)	76 (27.4)	78 (35.3)	3.08 (1.79–5.31)	< .001	2.76 (1.14–6.66)	**.024**
40–49	105 (21.1)	49 (17.7)	56 (25.3)	3.43 (1.91–6.16)	< .001	3.24 (1.12–9.42)	**.031**
50 +	135 (27.1)	74 (26.7)	61 (27.6)	2.47 (1.41–4.32)	.001	3.35 (1.35–8.27)	**.009**
*Sexual identity*							
Gay	455 (91.4)	245 (88.4)	210 (95.0)	Ref		Ref	
Bisexual/queer/other identity	43 (8.6)	32 (11.6)	11 (5.0)	0.40 (0.20–0.82)	.012	0.14 (0.05–0.42)	** < .001**
*State or territory*							
New South Wales	196 (39.4)	98 (35.4)	98 (44.3)	Ref		Ref	
Victoria	121 (24.3)	62 (22.4)	59 (26.7)	0.95 (0.60–1.50)	.83	1.39 (0.68–2.82)	.369
Queensland	73 (14.7)	48 (17.3)	25 (11.3)	0.52 (0.30–0.91)	.022	1.09 (0.44–2.75)	.847
Other jurisdictions	108 (21.7)	69 (24.9)	39 (17.6)	0.57 (0.35–0.92)	.02	0.82 (0.37–1.82)	.629
*Residential location*							
Capital city	360 (72.3)	190 (68.6)	170 (76.9)	Ref		Ref	
Other city/regional/rural or remote area	138 (27.7)	87 (31.4)	51 (23.1)	0.66 (0.44–0.98)	.04	0.88 (0.46–1.70)	.709
*Country of birth*							
Australia	335 (67.3)	199 (71.8)	136 (61.5)	Ref		Ref	
Elsewhere	163 (32.7)	78 (28.2)	85 (38.5)	1.59 (1.09–2.32)	.015	0.88 (0.51–1.51)	.641
*Education level*							
High school/Trade certificate	174 (34.9)	108 (39.0)	66 (29.9)	Ref		Ref	
University degree	324 (65.1)	169 (61.0)	155 (70.1)	1.50 (1.03–2.19)	.034	1.21 (0.65–2.26)	.547
*Employment status*							
Full-time	326 (65.5)	165 (59.6)	161 (72.9)	Ref		Ref	
Part-time	73 (14.7)	50 (18.1)	23 (10.4)	0.47 (0.27–0.81)	.006	1.03 (0.41–2.61)	.95
Student/unemployed/other	99 (19.9)	62 (22.4)	37 (16.7)	0.61 (0.39–0.97)	.037	0.89 (0.39–2.00)	.771
*Income level (AUD)*							
Less than $40,000	74 (14.9)	52 (18.8)	22 (10.0)	Ref		Ref	
$40,000 − $79,999	129 (25.9)	80 (28.9)	49 (22.2)	1.45 (0.78–2.67)	.236	0.66 (0.21–2.03)	.466
$80,000 − $120,000	127 (25.5)	68 (24.5)	59 (26.7)	2.05 (1.12–3.77)	.021	1.08 (0.31–3.77)	.903
More than $120,000	142 (28.5)	60 (21.7)	82 (37.1)	3.23 (1.77–5.88)	< .001	0.90 (0.23–3.53)	.876
Prefer not to say	26 (5.2)	17 (6.1)	9 (4.1)	1.25 (0.48–3.23)	.643	0.55 (0.11–2.83)	.475
*Relationship length with regular male partner(s)*							
Less than 1 year	64 (12.9)	48 (17.3)	16 (7.2)	Ref		Ref	
1–2 years	54 (10.8)	35 (12.6)	19 (8.6)	1.63 (0.74–3.61)	.229	2.09 (0.54–8.03)	.285
2–5 years	96 (19.3)	55 (19.9)	41 (18.6)	2.24 (1.12–4.48)	.023	2.21 (0.73–6.72)	.161
More than 5 years	284 (57.0)	139 (50.2)	145 (65.6)	3.13 (1.70–5.77)	< .001	1.27 (0.42–3.81)	.673
*Relationship type*							
Monogamous	173 (34.7)	157 (56.7)	16 (7.2)	Ref		Ref	
Non-monogamous	325 (65.3)	120 (43.3)	205 (92.8)	16.76 (9.56–29.39)	< .001	4.93 (2.01–12.10)	** < .001**
*Relationship agreement*							
Implicit agreement	103 (20.7)	65 (23.5)	38 (17.2)	Ref		Ref	
Explicit agreement	308 (61.8)	154 (55.6)	154 (69.7)	1.71 (1.08–2.71)	0.022	2.41 (1.15–5.05)	**.02**
No rules or agreement	87 (17.5)	58 (20.9)	29 (13.1)	0.86 (0.47–1.56)	.609	1.19 (0.47–2.98)	.716
*Regular male partner status incl. PrEP use and viral load*							
HIV-negative	266 (53.4)	205 (74.0)	61 (27.6)	Ref		Ref	
HIV-negative on PrEP	156 (31.3)	26 (9.4)	130 (58.8)	16.80 (10.10–27.95)	< .001	7.88 (3.89–15.96)	** < .001**
HIV-positive	43 (8.6)	20 (7.2)	23 (10.4)	3.86 (1.99–7.51)	< .001	3.92 (1.67–9.21)	**.002**
Don't know/untested	33 (6.6)	26 (9.4)	7 (3.2)	0.90 (0.37–2.19)	.824	2.07 (0.60–7.10)	.25
*No. of male partners in last 6 months*							
None	38 (7.6)	34 (12.3)	4 (1.8)	Ref		Ref	
1–5	271 (54.4)	193 (69.7)	78 (35.3)	3.44 (1.18–10.00)	.024	1.46 (0.32–6.77)	.627
6–10	80 (16.1)	30 (10.8)	50 (22.6)	14.17 (4.57–43.88)	< .001	1.17 (0.22–6.33)	.852
> 10	109 (21.9)	20 (7.2)	89 (40.3)	37.82 (12.05–118.74)	< .001	2.57 (0.44–15.09)	.296
*Sex with casual male partners (last 6 months)*							
No regular partners/no anal sex	208 (41.8)	179 (64.6)	29 (13.1)	Ref		Ref	
Consistent condom use	44 (8.8)	31 (11.2)	13 (5.9)	2.59 (1.21–5.52)	.014	1.07 (0.33–3.42)	.909
Any condomless sex	246 (49.4)	67 (24.2)	179 (81.0)	16.49 (10.18–26.71)	< .001	4.96 (2.11–11.70)	** < .001**
*Sex with regular male partners (last 6 months)*							
No casual partners/no anal sex	110 (22.1)	80 (28.9)	30 (13.6)	Ref		Ref	
Consistent condom use	30 (6.0)	23 (8.3)	7 (3.2)	0.81 (0.32–2.09)	.665	0.40 (0.10–1.50)	.172
Any condomless sex	358 (71.9)	174 (62.8)	184 (83.3)	2.82 (1.77–4.50)	< .001	0.60 (0.26–1.38)	.229
STI diagnosis in last 12 months	93 (18.7)	19 (6.9)	74 (33.5)	6.84 (3.97–11.77)	< .001	2.18 (1.03–4.63)	**.042**
Knows PrEP users	400 (80.3)	189 (68.2)	211 (95.5)	9.82 (4.96–19.45)	< .001	5.04 (2.05–12.39)	** < .001**
Knows people living with HIV	317 (63.7)	154 (55.6)	163 (73.8)	2.24 (1.53–3.29)	< .001	0.85 (0.45–1.60)	.612
Drug-enhanced sex in last 6 months	65 (13.1)	18 (6.5)	47 (21.3)	3.89 (2.18–6.92)	< .001	0.98 (0.45–2.12)	.95

Bolded values indicate statistical significance at *p* < ..05 (two-tailed)

Model *X*^2^(36) = 163.0, *p* < .001; McFadden’s pseudo *R*^2^ = .50. ROC = .93. Hosmer–Lemeshow *X*^2^(8) = 5.3, *p* = .73. *CAIC* = condomless anal intercourse with casual partners; *CAIR* = condomless anal intercourse with regular partners

### PrEP use

PrEP use was measured with the item ‘Are you currently taking PrEP?.’ Participants who selected any of these options (daily/most days; on demand, 2–1-1; periodic PrEP) were coded as current PrEP users.

### Current Relationships

The main analysis uses a sub-sample of non-HIV-positive men who indicated they were in a relationship with a male partner at the time of the survey. Current relationships with male partners were measured with the item ‘Do you currently have a regular male partner (or partners)?,’ with the response categories including boyfriend, partner or husband; fuck buddy (or fuck buddies); none of the above. Participants who selected ‘Boyfriend, partner or husband’ were asked about the length of their relationship and characteristics of any relationship agreement (i.e., monogamous vs. non-monogamous; implicit vs. explicit vs. no agreement).

### Relationship Type and Relationship Agreement

Participants were also asked ‘How would you describe your sexual relationship with your current regular male partner?,’ with the response options ‘We are monogamous (neither of us has casual sex),’ ‘We have an open relationship – my partner or I have casual sex,’ and ‘I have several regular male partners.’ The first category was coded as monogamous, the other two categories as non-monogamous. In relation to how agreements were reached, participants were asked ‘Do you and your regular male partner have clear rules (an agreement) about casual sex?.’ Responses were coded as ‘explicit agreement’ (‘Yes, we have discussed them’), ‘implicit agreement’ (‘Yes, but the rules are unspoken/assumed’), or ‘no agreement’ (‘No, we don’t have rules or an agreement’).

### Partner HIV Status and use of antiretrovirals

Participants were asked about their partner’s HIV status and their partner’s use of antiretrovirals (i.e., PrEP or HIV treatment, where appropriate). Participants who were in relationships and taking PrEP were asked if their partner knew they were taking PrEP (yes vs. no). Participants with more than one regular partner were instructed to choose the one with whom they spent the most time when answering the questions.

### Drug-enhanced Sex

Drug-enhanced sex was measured with the item ‘In the last 6 months, how often have you used drugs for the purpose of sex (e.g., MDMA, GHB, Crystal)?,’ with response categories including every week; at least monthly, once or twice; never. The first three categories were coded as any ‘drug-enhanced sex in the last 6 months.’

### Statistical Analyses

The analyses included HIV-negative and untested (non-HIV-positive) participants. HIV-positive participants were excluded. Pearson’s chi-squared tests were used to explore potential differences in the characteristics, sexual practices and other risk indicators by relationship status and PrEP use. Four groups were compared for each possible combination of PrEP use (using PrEP vs. not) and relationship status (in a relationship vs. not). Bivariate comparisons were also made for PrEP users by relationship status (in a relationship vs. not), and separately, for men in relationships by PrEP use (using PrEP vs. not). The latter comparison (of men in relationships by PrEP use) formed the basis for the multivariate analysis. This explored differences in the characteristics and reported sexual behavior between men in relationships who were taking PrEP compared to other men in relationships who were not taking PrEP. Variables were chosen based on the study aims (e.g., relationship type) and previous research that has identified factors associated with PrEP use, for example, socioeconomic status and sexual practices (MacGibbon et al., 2020, 2021). We used binary logistic regression to compare characteristics associated with PrEP use for men in relationships. Variables for which there were statistically significant differences at a bivariate level were then block entered into a multivariate logistic regression model to identify independent relationships with PrEP use. Statistical assumptions and model diagnostics for logistic regression were assessed, none of which were violated, and there were no missing data. We report unadjusted and adjusted odds ratios (OR and aOR) and 95% confidence intervals (CI). Statistical significance was set at *p* < .05 (two-tailed). Analyses were conducted using Stata version 16.1 (StataCorp, College Station, TX).

## Results

A total of 1,280 participants completed the survey in 2021. The median age was 38 years (IQR = 30–52). Most identified as gay (80.8%), were Australian born (71.6%), university educated (58.4%), and lived in the capital city of their state or territory (70.1%). Most participants lived in one of Australia’s three most populous states: New South Wales (37.7%), Victoria (24.8%), or Queensland (16.3%). Most participants reported full-time employment (60.2%). Most participants were HIV-negative (82.7%), with smaller proportions of untested (9.8%) and HIV-positive (7.4%) participants. Two thirds of the sample had been tested for HIV (69.2%) or other sexually transmissible infections (66.6%) within the 12 months preceding the survey. Less than half (42.4%; *n* = 543) of those surveyed were in a relationship with a male partner at the time of the survey. Among these 543 participants, 252 (46.4%) were HIV negative and were not taking PrEP at the time of the survey, 219 (40.3%) were HIV negative and were taking PrEP, 45 (8.3%) were HIV positive (nearly all of whom had an undetectable viral load), and 27 (5.0%) did not know their HIV status (all of whom had never been tested for HIV).

The sub-sample of non-HIV-positive men who were in relationships with male partners included 498 participants (42.0% of the total sample). A small number of participants in this group were also in relationships with female partners (*n* = 2), or had regular female sex partners (*n* = 13). There were 221 participants who were in relationships with other men and were taking PrEP (44.4% of GBM in relationships), of whom, most (88.7%) indicated that their partner knew they were taking PrEP; and of this group, most (73.0%) had explicit (discussed) relationship agreements. In contrast, of the participants who had not disclosed their PrEP use to their partner, only 44.0% had an explicit relationship agreement. There were no significant differences between participants who had disclosed or discussed their PrEP use with their male partner compared to those who had not in terms of age, residential location (state or regional vs. city), relationship length, or having a relationship agreement that specified monogamy or non-monogamy (all *p* > .05).

Table 1 shows demographic characteristics, sexual practices and risk indicators of non-HIV-positive participants (*n* = 1,185). Results were stratified by four comparison groups, which represented each possible combination of PrEP use (using PrEP vs. not) and relationship status (in a relationship vs. not). Overall, the groups differed on most measures (all *p* < .001, see Table 1). However, most of these differences were between PrEP users and non-PrEP-users (e.g., PrEP users reported more sexual partners, condomless sex with casual partners, recent HIV testing and STI diagnoses than non-users). We therefore focused on the practices of PrEP users as one group and then assessed differences between PrEP users in relationships and PrEP users not in relationships.

### PrEP use and Sexual Practices of PrEP Users

Over one third of participants (36.9%, *n* = 472) was taking PrEP at the time of the survey. The median age was 38.5 years (IQR = 31.5–50.0), and most (89.6%) identified as gay. One third (33.1%) of PrEP users reported having one to five male sexual partners in the six months preceding the survey; one quarter (25.0%) had six to 10 partners; more than one third (39.0%) had more than 10 partners, while a small proportion (3.0%) reported no partners. Most PrEP users (81.8%) reported having condomless anal intercourse with casual partners (CAIC) within the six months preceding the survey. Most PrEP users (77.3%) were taking PrEP daily, with the remaining proportions taking PrEP ‘on demand’ (19.7%) or daily for limited periods of time (‘periodic PrEP’; 4.0%). A fifth (22.3%) of PrEP users had been taking PrEP for under a year; a similar proportion (22.7%) for one to two years; more than one third (36.4%) for two to four years; and the remaining proportion (18.6%) for more than four years.

### Bivariate Comparisons of PrEP Users by Relationship Status

We compared whether PrEP users in relationships differed from other PrEP users who were not in a relationship, specifically, in terms of their PrEP use, sexual practices and indicators of risk. The frequencies and proportions for these comparisons, except the way PrEP was taken, are shown in Table 1 (see PrEP users). In short, there were no significant bivariate differences between PrEP users in relationships compared to other PrEP users in terms of the number of male sexual partners (*X*^2^(3, 472) = 3.62, *p* = .31), or condom use with casual partners (*X*^2^(2, 472) = 0.73, *p* = .69). There were no differences in the way PrEP was taken, with PrEP users in and out of relationships similarly likely to use PrEP daily (77.3% and 75.1%, respectively), on-demand (19.5% and 19.1%), or periodically (3.2% and 5.0%; *X*^2^(2, 472) = 1.02, *p* = .60). Nor were there differences between PrEP users in relationships compared to other PrEP users as to whether they ‘often’ or ‘always’ used risk reduction strategies when they had condomless sex, specifically, seeking out partners of the same HIV status (serosorting; *X*^2^(1, 472) = 3.38, *p* = .07), seeking out PrEP users (PrEP sorting; *X*^2^(1, 472) =0 .01, *p* = .97), or seeking out partners with an undetectable viral load (UVL sorting; *X*^2^(1, 472) = 0.17, *p* = .69). Lastly, PrEP users were similarly likely to report participating in drug-enhanced sex, regardless of relationship status (*X*^2^(1, 472) = 0.01, *p* = .97).

### Multivariate Analysis of PrEP use by GBM in Relationships

Table 2 shows the sample characteristics and factors associated with PrEP use versus non-use among the sub-sample of non-HIV-positive men in relationships (*n* = 498). Bivariate statistical differences were observed between the groups for all variables. The multivariate analysis showed that PrEP use among partnered men was independently associated with older age, specifically being aged between 30 and 39 (*aOR* = 2.76, 95% CI [1.14, 6.66]), 40 and 49 (*aOR* = 3.24, 95% CI [1.12, 9.42]), and being older than 50 years (*aOR* = 3.35, 95% CI [1.35, 8.27]), compared to those aged under 30 years. Bisexual, queer and other MSM in relationships were less likely to use PrEP than gay-identified participants (*aOR* = 0.14, 95% CI [0.05, 0.42]). Participants in non-monogamous relationships were more likely to use PrEP (*aOR* = 4.93, 95% CI [2.01, 12.10]), as were participants who had spoken (explicit) relationship agreements with their partners (*aOR* = 2.41, 95% CI [1.15, 5.05]). PrEP use was more likely among participants who had HIV-negative partners taking PrEP (*aOR* = 7.88, 95% CI [3.89, 15.96]), participants who had partners living with HIV (*aOR* = 3.92, 95% CI [1.67, 9.21]), those who had had any condomless sex with casual partners in the six months preceding the survey (*aOR* = 4.96, 95% CI [2.11, 11.70]), a diagnosis of an STI other than HIV in the previous year (*aOR* = 2.18, 95% CI [1.03, 4.63]), and among participants who knew at least one other person taking PrEP (*aOR* = 5.04, 95% CI [2.05, 12.39]).

## Discussion

Using national, cross-sectional survey data, we compared non-HIV-positive gay and bisexual men by PrEP use and relationship status. We found that being in a non-monogamous relationship and having a spoken (explicit) agreement about non-monogamy were independently associated with PrEP use among GBM in relationships. We also found that PrEP users in and out of relationships had similar sexual practices. Our results suggest that although gay and bisexual men in relationships may be suitable candidates for PrEP, open communication to form a relationship agreement may impact the likelihood of PrEP use. If unaddressed, the difficulties that many GBM in relationships experience discussing non-monogamy could impede PrEP adoption among those who could otherwise benefit from its use.

The PrEP users in our sample were similar to other samples of GBM using PrEP in terms of sexual practices and risk indicators (Hammoud et al., 2019; Traeger et al., 2018), and being in a relationship did not seem to make a noticeable difference. There were, however, differences between participants in relationships who used PrEP compared to those in relationships who did not use PrEP. The PrEP users in relationships were much more likely than non-users to report condomless sex with casual partners in the six months preceding the survey (over 80% of PrEP users vs. 24% of non-users). They were also more likely to have been diagnosed with a sexually transmissible infection other than HIV in the 12 months preceding the survey (34% vs. 7% of non-users). The higher proportion of STI diagnoses among PrEP users likely reflects the greater number of sexual partners that PrEP users reported, as well as the greater likelihood of condomless sex with casual partners and greater frequency in STI testing. PrEP users were also more likely to report having an HIV-positive partner (nearly all of whom had an undetectable viral load), which aligns with previous research (MacGibbon et al., 2020). Although it is not clear from these data whether PrEP users perceived there to be any risk of HIV from their primary partners, it is unlikely that these participants were only taking PrEP for protection from HIV within the relationship given that most HIV-positive partners had undetectable viral loads, and nearly all PrEP users with HIV-positive partners were in non-monogamous relationships (91%, *n* = 21/23). Lastly, in terms of sociodemographic factors, older men in relationships were more likely to use PrEP, while non-gay-identifying men (bisexual, queer, other identities) were less likely to use PrEP. At a bivariate level, PrEP use was also associated with higher income, education and full-time employment, but these were not independently associated with PrEP use among GBM in relationships.

Previous research has shown how relationship agreements are important for HIV prevention among men in relationships (see Rios-Spicer et al., 2019, for a review). For example, relationship agreements were central to traditional negotiated safety agreements–particularly in Australia-in which GBM agreed to either not have casual sex or to use condoms with all casual partners, after explicit discussion, testing and knowledge of HIV status (Crawford et al., 2001; Kippax et al., 1997). The current study shows that relationship agreements remain important for HIV-negative men in relationships who adopt PrEP, with PrEP users being more likely to have a non-monogamous and an explicit agreement. Previous research has also found that relationship agreements allowing condomless sex with casual partners have become much more common among GBM in the PrEP era (MacGibbon et al., 2020). So, while relationship agreements remain important for HIV prevention for GBM in relationships, the types of agreement that are formed and the sex permitted in and outside the relationship appear to have changed.

We also found that taking PrEP is a shared experience for many PrEP users in relationships. For example, PrEP users in relationships were much more likely to have a partner who also used PrEP (59% of PrEP users vs. 9% of non-users). This is consistent with previous research that found GBM in relationships who initiate PrEP are more likely to also have a partner who uses PrEP (Bavinton et al., 2021). Relatedly, most PrEP users (89%) in our sample reported that their partner knew they were taking PrEP. Lastly, prior Australian research has found that knowing other PrEP users drives PrEP uptake within social networks of GBM (Holt et al., 2019). If PrEP is perceived as socially normative, then this may prompt spoken agreements about its use among men in relationships. In the present study, nearly all PrEP users knew at least one person taking PrEP (96% of PrEP users vs. 68% of non-users). It is likely, however, that the way GBM form and monitor relationship agreements has changed as GBM adopt PrEP. For example, partners may find it easier to check on and encourage PrEP use than to verify that one’s primary partner is maintaining consistent condom use with casual partners, and maintaining sexual health and dealing with STIs may be easier, as this tends to occur at routine appointments to get new PrEP prescriptions. For these reasons, an agreement is likely to be more important at the stage where one or both partners decide to initiate PrEP (or acknowledge its existing use). Qualitative research would be ideal to explore the dynamics of relationship agreements that incorporate PrEP.

We believe this is the first study to report that PrEP users in relationships are similar in sexual behavior and other practices to PrEP users who are not in relationships. Although we thought it plausible that PrEP users in relationships may report fewer sexual partners and less condomless sex with casual partners, this was not evident in our sample. PrEP users in and out of relationships also had similar PrEP use patterns; that is, they were not more likely to be on-demand or periodic PrEP users. These findings provide important information for researchers, clinicians and educators who work with gay and bisexual men. Although PrEP users in relationships may need additional support to form relationship agreements that incorporate PrEP, or to negotiate non-monogamy, our results indicate there is no evidence to suggest men in relationships have less need for PrEP than other GBM who meet PrEP suitability criteria. A small group of participants were of specific concern and would benefit from more effective HIV prevention. Nearly a quarter of men in relationships who were not using PrEP (approximately 6% of the overall sample) also reported condomless sex with casual partners, the highest risk practice for HIV transmission in Australia (Down et al., 2017; Holt, Broady, et al., 2021). Clinicians should ask GBM in relationships about sex outside their relationship (if any) and encourage and support these men to consider more effective prevention options, like PrEP. Although the Australian PrEP guidelines encourage clinicians to take routine sexual histories (ASHM, 2019), future guidelines could include specific information about GBM’s relationships (beyond serodiscordance), to highlight the continued importance of sexual history taking with GBM in relationships. For example, clinicians should be familiar with the prevalence of non-monogamy among GBM in relationships (65% in this sample), the decline in the proportion of GBM with negotiated safety agreements (Mao et al., 2020), and the increase in the proportion of GBM in relationships who have condomless casual sex in Australia (MacGibbon et al., 2020). This information would assist clinicians to build rapport with GBM in relationships and more accurately identify HIV risk.

We acknowledge the limitations of the analysis. Data were collected during April–June 2021, and the survey recall periods of 6–12 months included periods of COVID-19 restrictions and lockdowns, which may have affected participants’ sexual behavior and relationships. Although our sample appeared to report levels of sexual behavior consistent with previous rounds (MacGibbon et al., 2021), and relatively high levels of condomless sex with casual partners, other research found that PrEP use and sexual behavior were reduced during COVID-19 restrictions (Hammoud et al., 2020, 2021). It is also possible that fewer relationships were formed during this period; notably, 14% of our sample were in relationships of less than two years duration compared to 36% in a recent, pre-COVID-19 analysis (MacGibbon et al., 2020). We did not assess whether participants who had spoken (explicit) agreements with their partner had kept to their agreement, or if they had extended or breached their agreement (such as by having condomless sex with casual partners when their agreement was to use condoms). We also did not assess the specific characteristics of participants’ agreements, for example, what type of sex was allowed and with whom, and how breaches were supposed to be managed. As noted above, we believe that qualitative research would be valuable to assess the characteristics of PrEP users’ relationship agreements and the strategies they use to form and monitor agreements. Lastly, a representative sample of GBM in Australia would likely include a broader age range, more bisexual men, and more residents from regional areas than we achieved in our study (Grulich et al., 2014).

## Conclusion

Our findings highlight that having a spoken (explicit) relationship agreement about non-monogamy is an important condition related to PrEP use among GBM in relationships. Relationship agreements continue to be important in the biomedical prevention era, even if the characteristics of those agreements appear to have changed and gay and bisexual men appear to have moved beyond solely negotiating condom use and non-use. Clinicians and educators should continue to support gay and bisexual men to establish or renegotiate effective relationship agreements that incorporate one or more HIV prevention strategies, and which meet the partners’ sexual and emotional needs.

## Data Availability

Not applicable.
